# Lying-In Hospital, Dublin, October 11th, 1858

**Published:** 1859-02

**Authors:** W. A. McPheeters


From the N. O. Medical & Surgical Journal.

*Lying-in Hospital, Dublin, October* 11*th*, 1858.

Dr. Stanford Chaille—*Dear Doctor*:—I have been intending for some time past to send you some account of the practice, etc., of the Dublin Lying-in Hospital, but have been prevented by various circumstances from doing so before this. This Hospital was founded more than a century ago, and is the largest of the kind in the United Kingdom. It contains one hundred and thirty beds, fifteen of which are reserved for chronic diseases of the uterus and its annexes. About two thousand women are confined here annually, making an average of five *per diem*.

The medical staff consists of a master, Dr. McClintock, and two assistants, all of whom reside in the Hospital. The master is elected by the Board of Governors, and holds his office for seven years. The assistants each pay five hundred dollars *per annum* for the privileges they enjoy. The *interne* pupils pay one hundred dollars for six months, or half that amount for three months, besides their board. The advantages here are very good, though not as much so as could be wished. Students are not permitted to perform operations, however well qualified they may be, but can only look on while they are performed by the master or his assistants. At Vienna, I am told, students enjoy the privilege of applying the forceps, or making version when necessary.

3

The ventilation of this Hospital is very good, and every attention is paid to cleanliness; but notwithstanding every precaution, there are occasional epidemics of puerperal fever. In each ward is a couch on which the patient is delivered, and two hours afterwards, she is removed to her bed. A purgative is usually given on the second day after delivery, and if the patient goes on well, she is allowed to sit up on the fifth day, and is discharged from the Hospital on the eighth day. Prolapsus uteri appears to be very common among the lower classes of Dublin, which is doubtless owing, in many cases, to getting up too soon after confinement.

Dr. McClintock lays great stress upon the use of the binder after delivery, to keep the uterus firmly contracted, and to diminish the size of the abdomen. In the Paris Hospitals, the binder is considered useless, and is not usually applied. The patients here are always delivered on the left side, not only in natural labors, but also in all operations. In Paris they are always delivered on the back. In natural cases I think the side position is preferable, but when an operation is to be performed, I think it is much more convenient to place the patient on her back, with the hips drawn over the edge of the bed.

In all obstetrical operations here, chloroform is usually given, unless there is some special contradiction. It is especially useful when it is necessary to make version, or to introduce the hand to bring away a retained placenta.

An interesting case occurred a few days since showing the advantages of chloroform. It was a case of retained placenta caused by spasmodic contraction of the cervix uteri. After waiting four hours without result, Dr. McClintock decided to administer chloroform, and to introduce the hand; gradually dilate the os uteri and bring away the placenta. The patient was with considerable difficulty brought under the full influence of the chloroform, and on introducing the hand into the vagina, the os uteri was found to be dilated, and the placenta extruding, so that it was easily extracted, without introducing the hand into the uterus, which may justly be considered one of the most dangerous operations in obstetrics.

The only forceps used in this Hospital are the straight forceps, but with longer blades than the ordinary short forceps. The following are considered indispensable conditions in order to render the forceps applicable: 1st. "That the child be alive, or at least that there is no reasonable ground for supposing it to be dead." When the child is ascertained to be dead, the perforator and crotchet are used, being considered as less dangerous to the mother than the application of the forceps. The stethoscope renders most valuable aid in ascertaining the condition of the fœtus. If the fœtal heart is heard in the early stage of the labor, and afterwards the most careful examinations fails to detect it, the death of the fœtus may be considered as almost certain if not absolutely so. Hence it is an established rule of the Hospital to ascertain if the fœtal heart is audible, when the patient enters, so that if the labor becomes difficult, and instrumental aid is required, it can be ascertained with almost absolute certainty whether the child be alive or dead. 2d. "That the head has remained stationary within reach of the forceps, for six hours at least." This rule, of course, is not adhered to, when there exists any pressing complication, such as hæmorrhage or convulsions, etc. 3d. "That the membranes be ruptured and the os uteri fully dilated." 4th. "That the head of the child be so circumstanced that the ear can be distinctly felt, without the use of any force or violence on the part of the examiner. Dr. Rigby of London, does not approve of this plan of feeling for the ear. In his "system of midwifery," he very justly remarks: "The blades should always, if possible, be applied one on each side of the head, the position of which must be determined by the direction of the fontanelles and sutures, *not by feeling for the ear*, as is usually recommended in this country. The ear can seldom be reached without causing a good deal of pain, even under the most favorable circumstances,,' &c., &c. 5th. "That the state of the soft parts be such as denotes the absence of inflammation; in other words, that they be free from undue heat, dryness, tumefaction, or morbid sensibility." These views are extracted from Hardy and McClintock's report of the Hospital practice.

When the above conditions are absent and instrumental aid is necessary, craniotomy is performed with the perforator and crotchet. The cephalotribe is never used—it being considered a dangerous and unnecessary instrument. The ergot of rye is used with great caution during labor, as experience has shown that it exerts a very injurious influence on the fœtus, unless it be born soon after the ergot has been given. In cases of inertia uteri, when the head presents and the os uteri is fully dilated, and stimulating enemata of salt and water have failed to excite efficient contractions, ergot, ʒss., is given and the dose repeated in twenty minutes if necessary. But if the child is not born within *two hours* after the administration of the ergot, the forceps is applied and the child speedily extracted, as the experience of this Hospital has been that the child was generally still-born, if not born within two hours after the ergot was given. Dr. McClintock is opposed to the administration of ergot during the third stage of labor, to produce the expulsion of the placenta. He says that if the placenta should happen to be morbidly adherent, the ergot might cause hour-glass contraction, render the operation of introducing the hand into the uterus to detach the placenta, much more difficult, and, consequently, more dangerous to the patient. It is the common practice here to push out the placenta by grasping the fundus uteri and making considerable pressure which is generally effective, if the placenta is detached.

We have recently had quite a number of cases of puerperal fever. The usual treatment consists in local depletion by means of leeches to the seat of tenderness; mercury and opium internally and mercurial inunctions to the abdomen. I cannot say that this treatment has been more successful than many others that have been recommended for the cure of this terrible malady. The muriated tincture of iron has recently been very much in vogue in London, as a remedy in puerperal fever. It was tried in several cases here, with varied success. Two cases appeared to be much benefitted, while two others died. I have not seen a sufficient number of cases to judge of its efficacy. It was not thought much of here, and was soon abandoned for the old treatment. One of the cases that appeared to be benefited at first, died after a month's illness from exhaustion produced by a phlegmonous inflammation of the calf of the leg. Twenty-four leeches w.re applied in the commencement of the attack, and then the muriated tincture of iron, (ten drops every two hours) was given. The other case appeared to be convalescing, when she was attacked with arthritis in the wrist. She was removed from the Hospital by her friends, before convalescence, so that I am not able to say whether she recovered or not.

To give you an idea of the advantages a student has for seeing obstetrical operations, I will mention that during three months that I have been *Interne* in this Hospital, there have heen eight forceps cases, three craniotomies, and two cases of version.

A very unnatural and interesting case of complicated labor occurred a few weeks since, the details of which may interest you: B. B., æt. thirty-seven, primipara, was admitted into the Hospital at noon on the 14th of September. She stated that at 9, A. M., on the 12th, she had been delivered of a dead child, at a village seven miles from Dublin. Her medical attendant discovered that there was another child in utero, and after waiting two days for its expulsion, advised her to come to the

Hospital. Accordingly, she was placed in a cab and brought in. On examination the os uteri was found to be half dilated, the membranes unbroken, the breech of a second child presenting, and the cord of the first hanging out of the vulva. In a short time the second child was born alive, and apparently of about eight months' development. It was now discovered that there was a third child presenting also by the breech. There being some hæmorrhage, it was deemed expedient to bring down the feet and hasten the delivery. The third child was also born alive. The uterus contracted, and the hæmorrhage ceased; but commenced again in about fifteen minutes, and the placentæ were still retained. There was no time to be lost; if the hæmorrhage was not speedily arrested, the patient would bleed to death. Although she was very much exhausted, it was deemed necessary to introduce the hand and bring away the placentæ without delay. This was accordingly done, giving her, at the same time, stimulants. There were two placentæ, both of which were partially morbidly adherent. The uterus now contracted firmly and all hæmorrhage ceased; but in spite of all efforts to sustain her vital powers she sank rapidly, and expired in a few hours after delivery, from the combined effects of exhaustion, arising from the protracted labor, the shock of delivery, and hæmorrhage. The pulse continued perceptible to the moment of dissolution. The great error committed by the "village doctor" in this case, was in not rupturing the membranes of the second fœtus, soon after the birth of the first, and before the os uteri had time to contract. Had this been done, the patient would have stood a much better chance of recovery, although the adherent placentæ and hæmorrhage would have rendered the prognosis very unfavorable.

The most remarkable feature of the case, however, was revealed at the autopsy: The uterus was very large, and on its anterior surface was an *enormous fibrous tumor*, seven or eight inches long and four or five wide. The woman stated on coming in, that she had perceived a tumor in the abdomen for about two years. This shows conclusively that organic disease of the uterus does not prevent conception, and it is not a little remarkable, that utero-gestation should have gone on almost to the full term with triplets, while, at the same time, there existed such a large amount of organic disease in the body of the uterus.

The children were in a healthy condition when taken from the Hospital.

Yours, very truly, W. A. McPHEETERS, M. D.

